# The Genus *Xanthagaricus*: An Updated Global Species Distribution and Phylogeny with the Description of Two New Species from Oman

**DOI:** 10.3390/jof8020173

**Published:** 2022-02-10

**Authors:** Moza Al-Kharousi, Shah Hussain, Marwa A. Al-Muharabi, Zahra Al-Shabibi, Dua’a Al-Maqbali, Abdullah H. Al-Balushi, Mohamed N. Al-Yahya’ei, Nadiya Al-Saady, Rethinasamy Velazhahan, Abdullah M. Al-Sadi

**Affiliations:** 1 Oman Animal and Plant Genetic Resources Center (Mawarid), Ministry of Higher Education, Research and Innovation, P.O. Box 515, Muscat 123, Oman; moza.alkharousi@trc.gov.om (M.A.-K.); Zahra.AlShabibi@oapgrc.gov.om (Z.A.-S.); duaa.almoqbali@mawarid.gov.om (D.A.-M.); abdullah.albalushi@mawarid.gov.om (A.H.A.-B.); mohamed.alyahyaei@mawarid.gov.om (M.N.A.-Y.); nadiya@mawarid.gov.om (N.A.-S.); 2 Department of Plant Sciences, College of Agricultural and Marine Sciences, Sultan Qaboos University, P.O. Box 34, Al Khoud 123, Oman; s.hussain2@squ.edu.om (S.H.); velazhahan@squ.edu.om (R.V.); 3 Sultan Qaboos Comprehensive Cancer Care & Research Center, P.O. Box 566, Al Khoud 123, Oman; marwa.almuharabi18@gmail.com

**Keywords:** 2 new taxa, Dhofar, phylogeny, Salalah, southern Oman

## Abstract

*Xanthagaricus* is a saprotrophic mushroom genus with small-sized basidiomata in the family Agaricaceae (Agaricales). Prior to this study, 26 species belonging to this genus have been described and published. In this study, we reported *Xanthagaricus* for the first time from Oman with the description of two new species. Basidiomata of the new species *Xanthagaricus appendiculatus* and *X. omanicus* were collected during the monsoon rains of summer in 2018 in the southern coastal region of Oman. Species descriptions are based on morphological and molecular characterization. Phylogenetic analyses based on internal transcribed spacer region (ITS1-5.8S-ITS2 = ITS) of the nuclear ribosomal DNA clustered the new species in the *Xanthagaricus* clade with strong statistical support. The new species *Xanthagaricus* *appendiculatus* can be distinguished from other species by its purplish pileus with umbonate disc and *X. omanicus* with the largest pileus (70–90 mm diameter) among the known species of the genus. A detailed description, photographs, line drawings, and a phylogenetic tree showing the position of both new species are provided. A dichotomous key to the known taxa of *Xanthagaricus* is proposed. Morphological comparisons of new species with known *Xanthagaricus* taxa are provided. Our observations highlight the diversity of *Xanthagaricus* and other lepiotaceous mushrooms in southern Oman and further document the need for additional systematic focus on the region’s fungi.

## 1. Introduction

**Figure 1:**
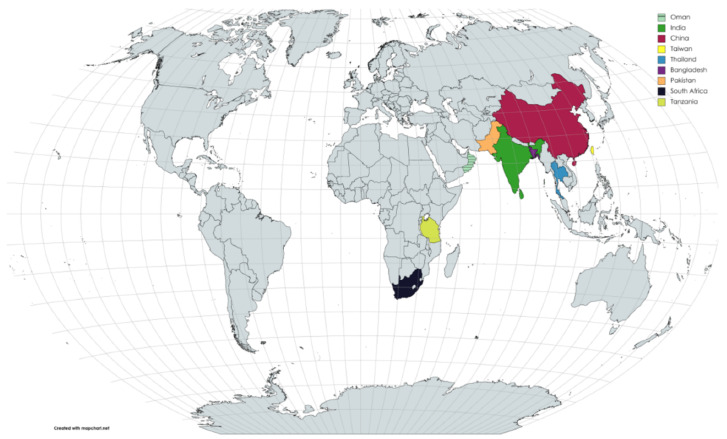
Global distribution of *Xanthagaricus* species. The countries where *Xanthagaricus* species have been reported are indicated in specific colors; the rest of the countries are in gray. (Source: MapChart, https://mapchart.net/world.html (accessed on 6 January 2022)).

The genus *Xanthagaricus* (Heinem.) Little Flower, Hosag., and T.K. Abraham is a small group of saprotrophic mushrooms in the family Agaricaceae [1,2,3]. Species of *Xanthagaricus* are characterized by small-sized to rarely medium-sized basidiomata with the squamulose pileus, the squamules comprised of hymeniform or pseudoparenchymatous cells, yellow to yellowish-brown basidiospores, and the absence of both pleurocystidia and clamp connections [1,2,4,5,6]. Members of *Xanthagaricus* are mostly distributed in equatorial paleotropics (Figure 1) [7]. To date, there are 26 described species in the genus *Xanthagaricus*; among these species, eleven species have been reported from India [1,2]; four species from Sri Lanka [1,2,8]; four species from Thailand [9,10,11]; two species from Africa [12,13]; three species from mainland China [5,14,15]; and one species each found in Bangladesh [5], Pakistan [6], and China’s Taiwan Island [7]. Previously, no species of *Xanthagaricus* and other lepiotaceous fungi have been reported from Oman.

Oman lies in the south-eastern part of the Arabian Peninsula [16]. The country has an area of about 309,500 square km with the coastline, covering approximately 3000 km from Musandam in the north to Dhofar in the south [17]. The land of Oman is mainly desert and valleys (82%), with mountains (15%) and coastal areas (3%) as well [18,19].

Oman has a subtropical dry and hot climate. The climatic conditions are varied according to geography. From October to April, the climate in Oman is mostly warm and sunny; daytime temperature rises up to 37 °C and is at night at about 17 °C [20]. From May to September, it is dry and hot in most parts of the country, and the temperature reaches up to 50 °C or above. The annual rainfall is about 100 mm [21]. Most regions of interior Oman are desert areas, known as the Rub’al-Khali [22]. The coastline has greater plant biodiversity [23]. The southern coastline of Oman, consisting of the Dhofar Governorate, is the most fertile area and receives plenty of monsoon rain from May to September [24,25]. The monsoon climate can influence the emergence of various macrofungi on different substrata, e.g., soil, log, stump, wood, leaf-litter, and dung.

The Sultanate of Oman has about 1390 species of vascular plants; most of these plants are endemic to the region [26]. Based on the most conservative approach of the 6:1 (six fungal species associated with one plant species) ratio used by Hawksworth [27], it is estimated that nearly 7000–8000 fungal species may occur in Oman. A checklist of the known fungi from Oman has been compiled, comprised of 318 fungal species belonging to 173 genera, mostly of Ascomycota [17]. It seems that only 4–5% of fungi of Oman are discovered, and the vast majority are unexplored. A similar case has been observed in northern Thailand, where fungal explorative expeditions showed that up to 96% of species in most agaric genera are new to science [28]. The reported fungi from Oman are mostly those causing diseases in different crops [29,30,31], and few of these belong to the macrofungi. It seems that the coastal region of Oman, which receives an ample amount of precipitation in summer, would be the hotspot of macrofungi. The fungal diversity studies in these areas will be particularly highly productive.

During the macrofungal exploration of Dhofar Governorate of Oman in 2018, some interesting specimens of lepiotaceous mushrooms were collected. According to morphological and molecular phylogenetic analyses, the specimens represent two new species of *Xanthagaricus* which are subsequently described in detail.

## 2. Materials and Methods

### 2.1. Sampling and Morphological Observations

**Figure 2:**
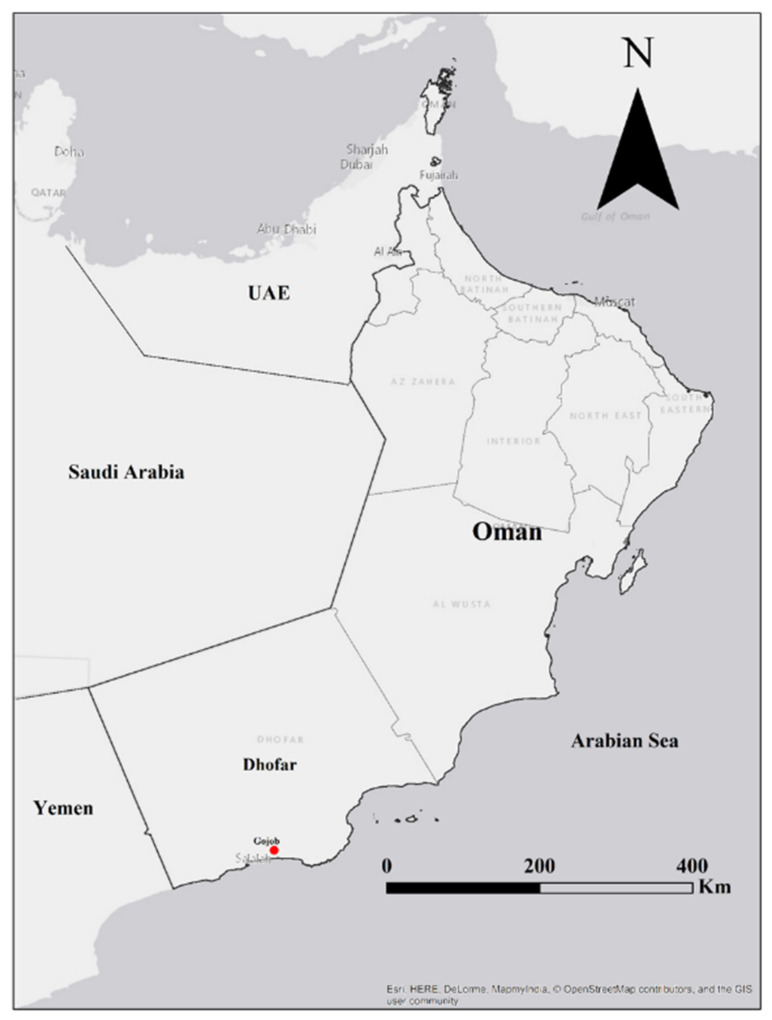
Map of Oman, the red dot represents the Gogob area, near Salalah in Dhofar Governorate, where the type specimens of *Xanthagaricus appendiculatus* and *X. omanicus* were collected. (Source: Esri, GIS User Community).

Specimens were collected from the Gogob area, around Salalah, Dhofar Governorate, Oman (Figure 2), during the monsoon rainy season in the month of September in the year 2018. The sampling area is arid with hot desert (BWh) climate using the latest Köppen climate map and classification [32,33]. However, the mountains of Dhofar are located in the monsoon belt. The area receives southwest monsoon from mid-June to mid-September every year, as part of the Inter Tropical Convergence Zone [34]. With such high precipitation in the monsoon, the vegetation of the area is mainly covered by deciduous trees [35].

Photographs of basidiomata were captured and labeled, and field notes were made. Munsell’s soil color charts were used for color determination [36]. Specimens examined in this study were submitted to SQUH herbarium, Department of Plant Sciences, CAMS, Sultan Qaboos University, Muscat, Oman.

For microscopic observations, slides were prepared in 5% aqueous KOH (*w*/*v*), followed by 1% aqueous Congo red (*w*/*v*). Microscopic characters, including the size and shape of basidiospores, basidia, cheilocystidia, caulocystidia, and pileipellis were studied under a light microscope (ECLIPSE Ni-U, Nikon Co., Ltd., Tokyo, Japan), with at least 20 structures measured in each instance. For basidiospores, measurements were done for 60 spores using 1000× magnification with a calibrated Nikon DS-Ri2 microscopic camera. Measurements of basidiospores are given as (a)b–c(d), where b–c includes a minimum of 90% of the measured values. Extreme values (a and d) are given in parentheses. The Q was calculated as the length/width ratio of spores, and av. Q is the mean length/width ratio of all basidiospores.

### 2.2. Molecular Identification

Extraction of the genomic DNA was done from dried specimens following a modified CTAB protocol [37]. The internal transcribed spacer region (ITS1-5.8S-ITS2 = ITS) of the nuclear ribosomal DNA was amplified using ITS1F/ITS4-B primer pair [37]. Polymerase chain reaction (PCR) was performed using PuReTaq^TM^ Ready-To-Go PCR beads, with 1.0 µL of each primer (10 µM/µL), 22 µL H_2_O, and 1 µL template DNA. PCR amplification was performed with 4 min initial denaturation at 95 °C, followed by 34 cycles of 50 s at 94 °C, 40 s at 54 °C, 50 s at 72 °C, and a final extension of 7 min at 72 °C followed the last cycle, following Al-Sadi et al. [38]. Purification of PCR products and sequencing with the same primers were carried out at ©Macrogen Inc. (238, Teheran-ro, Gangnam-gu, Seoul, Korea).

### 2.3. Phylogenetic Analyses

The ITS sequences were edited and aligned in BioEdit v7.2.5 [39], and homology searches were done at NCBI (National Center for Biotechnology Information), using BLAST (Basic Local Alignment Search Tool) [40]. For phylogenetic analyses, the ITS dataset was constructed from sequences mentioned in the most recent phylogenetic analyses of *Xanthagaricus* [5,6,9,10,11,14,15]. DNA sequences were aligned using Clustal X 2.1 [41]. *Chlorophyllum rhacodes* (Vittad.) Vellinga was selected as an outgroup taxon. The resulting alignment was submitted to TreeBASE (S29213).

Maximum Likelihood (ML) and Bayesian Inference (BI) methods were used for phylogeny. The BI phylogeny was estimated using BEAST version 1.8.4 [42] with an uncorrelated lognormal relaxed clock. A Birth-Death Incomplete Sampling speciation model tree was selected [43]. After that, selection of the best-fit model (TIM2 + I + G) was done using jModelTest2 [44]. The analyses were run from the BEAST on XSEDE tool on the Cipres Science Gateway [45]. The obtained log files were entered in Tracer [46]. Then, log files and trees files were combined in LogCombiner 1.8.2 [47]. TreeAnnotator 1.8.2 [47] was used to produce the Maximum Clade Credibility tree.

Maximum Likelihood analyses were run in RAxML-VI-HPC on XSEDE tool [45]. The best-fit model (TIM2 + F + I + G4) was selected using ModelFinder [48]. Node support was obtained with 1000 pseudoreplicates under the GTRCAT model. Branch support was calculated by 1000 bootstrap replicates.

For phylogenetic tree visualization, FigTree 1.4.2 [49] was used, and the tree was annotated using Adobe Illustrator CC2018.

## 3. Results

### 3.1. Molecular Phylogenetic Analyses

**Figure 3:**
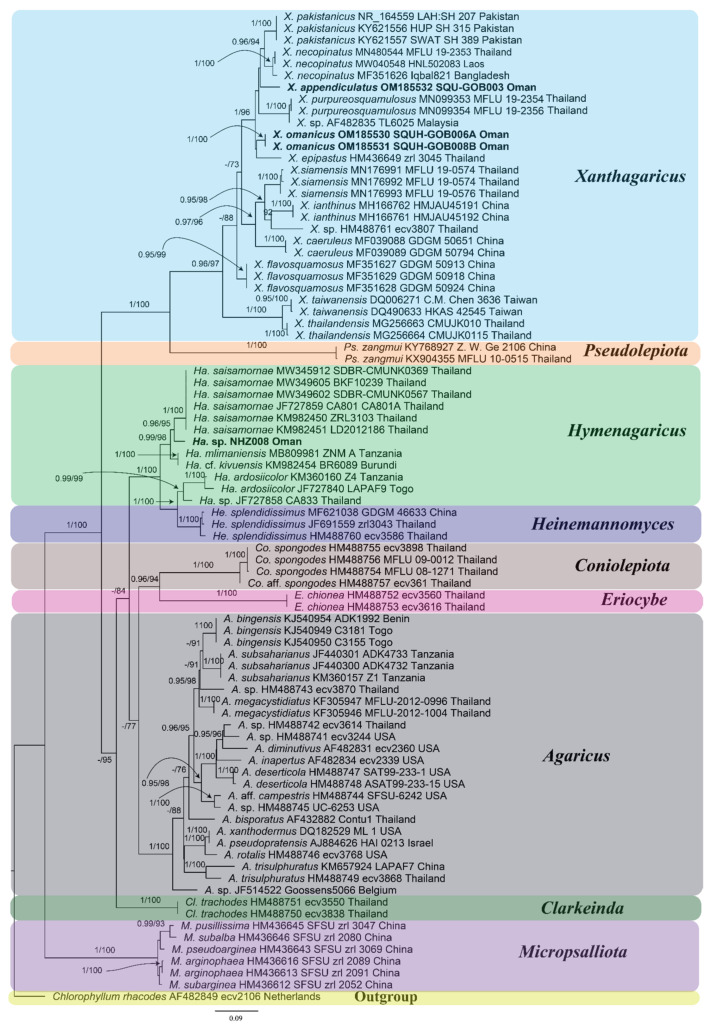
Phylogeny of *Xanthagaricus* and allied genera. This is the Maximum Likelihood (ML) phylogenetic tree with both the Bayesian posterior probabilities (PPs) and maximum likelihood bootstrap (BT) values indicated above the nodes, and each leaf represents the name of taxon, followed by ITS accession, voucher number, and country of origin; sequences representing the new species are in bold font.

A total of 84 ITS sequences were included for the phylogenetic reconstruction, and the dataset was 594 bp long after being trimmed. In the phylogeny, species of Agaricaceae clustered in the “*Agaricus* clade of Agaricaceae” and the *Micropsalliota* clade, as shown in (Figure 3), corresponding to the phylogeny of the family as shown by Vellinga et al. [3]. The *Agaricus* clade of Agaricaceae further comprised sub-clades: *Agaricus* L., *Clarkeinda* Kuntze, *Coniolepiota* Vellinga, *Eriocybe* Vellinga, *Hymenagaricus* Heinem., *Pseudolepiota* Z.W. Ge, and *Xanthagaricus*. Statistical support for both *Agaricus* clade of Agaricaceae and *Micropsalliota* clade was excellent in both analyses (BT 100%, PPs 1). Similarly, the statistical support for each sub-clade, each representing a genus, was excellent: BT 100% and PPs 1 for each *Agaricus*, *Clarkeinda*, *Coniolepiota*, *Eriocybe*, *Hymenagaricus*/*Heinemannomyces,* and *Pseudolepiota* clade. The representative genus *Xanthagaricus* recovered with strong statistical support (BT 90%; PPs 0.96). The two new species *X. omanicus* sp. nov. and *X. appendiculatus* sp. nov. nested in clade comprised of *X. epipastus* (Berk and Broome) S. Hussain, *X. necopinatus* Hosen, T.H. Li, and G.M. Gates, *X. pakistanicus* S. Hussain, Afshan, and H. Ahmad and *X. purpureosquamulosus* Sysouph., Thongkl, and K.D. Hyde.

### 3.2. Taxonomy

#### 3.2.1. *Xanthagaricus appendiculatus* Al-Sadi and S. Hussain, sp. nov.

**MycoBank: 842465.**

**Figure 4:**
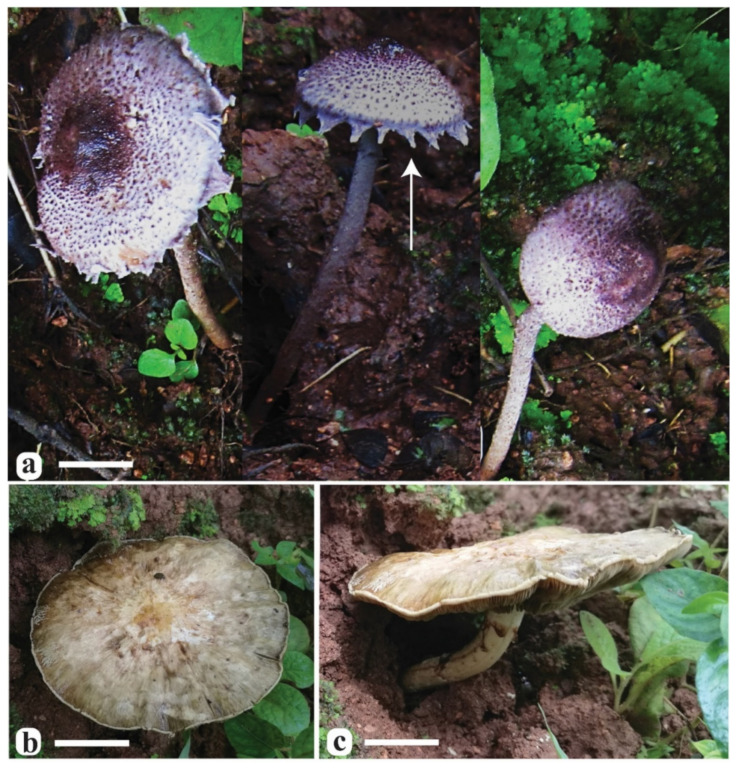
Basidiomata of *Xanthagaricus* species. (**a**) Holotype of *Xanthagaricus appendiculatus* (SQUH-GOO003); the arrow indicates the appendiculate pileus margin; (**b**,**c**) holotype of *Xanthagaricus omanicus* (SQUH-GOB006A). Scale bars: a = 10 mm, b, c = 20 mm.

**Figure 5:**
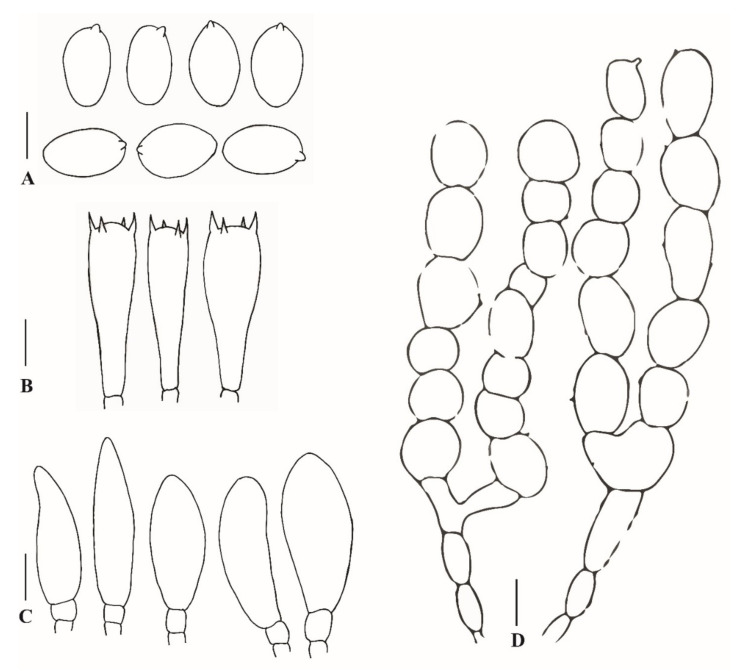
Anatomical features of *Xanthagaricus appendiculatus* (SQUH-GOO003). (**A**) Basidiospores; (**B**) Basidia; (**C**) Cheilocystidia; (**D**) Pileipellis. Scale bars: A = 5 µm, B, C = 10 µm.

**Etymology:** “*appendiculatus*” refers to the appendiculate veil remnants at the pileus margin of the new species (Figure 4a and Figure 5).

**Holotype:** Oman, Dhofar, Gogob, 17°20′98.9″ N and 54°08′77.6″ E, under the trees of *Anogeissus dhofarica*, 2 September 2018, SQUH collection GOO-003 (SQUH-GOO003) GenBank accession: ITS = OM185532.

**Diagnosis:** The diagnostic characteristics of the new species *Xanthagaricus appendiculatus* are convex to planoconvex pileus with umbonate disc, covered with reddish-purple squamules; basidiospores bluish to brownish, ellipsoid to amygdaliform, and comparatively larger (6.5–7.5 × 4.0–5.0 µm) than in its close relative *X. purpureosquamulosus*.

**Description:** Pileus 15–20 mm diam, at young stage parabolic, becoming hemispheric with age, at maturity convex to planoconvex, umbonate at the center; at first, pileus almost completely covered with smooth, dark reddish-purple (2.5 RP 3/4–2.5 RP 3/6) to very dark reddish-purple (2.5 RP 1/4–2.5 RP 1/6) pellicle, the pellicle disrupting during the pileus expansion, except at the umbonate center where it is retained as one or more large, dark purplish (5 P ¼–5 P 1/6) squamules, with small, radially arranged dark purplish (5 P ¼–5 P 1/6) squamules toward the margin, on pale violet (2.5 P 8/4) to violet (2.5 P 9/4) background; margin incurved, with pale violet (2.5 P 8/4) to violet (2.5 P 9/4) appendiculate triangular velar remnants. Lamellae free, pale bluish (5 B 6/2–5 B 8/2) to slightly dark-bluish (5 B 4/2–0 B 4/2) or ink-blue (5 PB ¼–10 B 1/6), broadly ventricose, sub-distant to nearly crowded with 3–4 tiers of lamellulae, lamellulae concolorous with lamellae, lamella edge slightly crenulate. Stipe 40–60 × 2–4 mm, equal, central, slightly curved, covered with pale-purple (5 P 8/2–5 P 6/2) to grayish-purple (7.5 RP 4/2–2.5 RP 3/2) squamules or fibrils; annulus not prominent, present as velar remnants at the apical zone of the stipe, concolorous to the pileus margin. In pileus, the context is white, up to 1 mm thick at the center; in stipe, hollow and concolorous with the surface. Odor and taste were not observed. Basidiospores (6.0)6.5–7.5(8.5) × (3.5)4.0–5.0(5.5) µm, on average 7.0 × 4.6 µm, Q = 1.5–1.7, av. Q = 1.6, ellipsoid to amygdaliform in side view, ellipsoid to ovoid in frontal view, thick-walled, smooth, pale-bluish to pale-brownish in 5% KOH, without germ pore. Basidia 19.0–22.0 × 6.5–7.5 µm, cylindrical to clavate, smooth, thin-walled, hyaline, 4-spored. Pleurocystidia absent. Cheilocystidia 26.0–33.0 × 7.0–8.5 µm, ventricose to clavate, hyaline, abundant. Pileipellis an irregular epithelium consisted of globose to subglobose (13.5–21.5 µm diam) cells in the upper layer, broadly ellipsoid to oblong cells (18–57 × 12–15 µm) in the lower layer and pale-brown to hyaline, up to 10 µm wide hyphae at the base of the epithelial layer, with some vacuolar pigments, near the septa in the basal epithelium. Stipe surface comprised of an irregular epithelium same as on pileus. Clamp connections were absent.

**Habitat and distribution:** Scattered in small groups, saprotrophic, on humus-rich soil with dead leaves and wood under trees of *Anogeissus dhofarica*. So far only known from southern Oman.

**Additional material examined:** Oman, Dhofar, Gogob, under the trees of *Anogeissus dhofarica*, 2 September 2018, SQUH collection GOO-003B (SQUH-GOO003B).

**Notes:***Xanthagaricus appendiculatus* is characterized macroscopically by the characteristic umbonate pileus disc, covered with reddish-purple squamules and microscopically by the morphology of its basidiospores and basidia. The basidia are elongated or cylindrical to clavate, 4-spored, measuring 19.0–22.0 × 6.5–7.5 µm.

Morphologically, the new species *Xanthagaricus appendiculatus* is similar to a recently described species from Thailand, *X. purpureosquamulosus* [11]. Both species share the same pileus characterictics, such as shape, radially arranged squamules on the surface, and umbonate center. However, the squamules are brownish in *X. pupureosquamulosus* as compared to the purplish squamules of *X. appendiculatus. Xanthagaricus pupureosquamulosus* has smaller basidiospores (4.5–6 × 3.5–4 µm), as well as basidia (10–12 × 5.5–7 µm) and cheilocystidia (10–16 × 5–8 µm). Both species can be differentiated on the basis of cheilocystidia shape; in *X purpureosquamulosus*, the cheilocystidia are clavate to ellipsoid, while in *X. appendiculatus*, these are consistently ventricose.

In our phylogenetic analyses, *X. appendiculatus* is shown to be related to *X. pakistanicus* and *X. necopinatus*. *Xanthagaricus pakistanicus* is recently described from Pakistan [6] and *X. necopinatus* from Bangladesh [5]. Our newly described species shares the pileus squamules characters with these species. *Xanthagaricus appendiculatus* can be separated from these taxa by the characteristic purplish pileal squamules, while the squamules are dark brown in *X. pakistanicus* and yellowish-brown in *X. necopinatus*.

#### 3.2.2. *Xanthagaricus omanicus* Al-Kharousi, Al-Sadi and S. Hussain, sp. nov. 

**MycoBank: 842466.**

**Figure 6:**
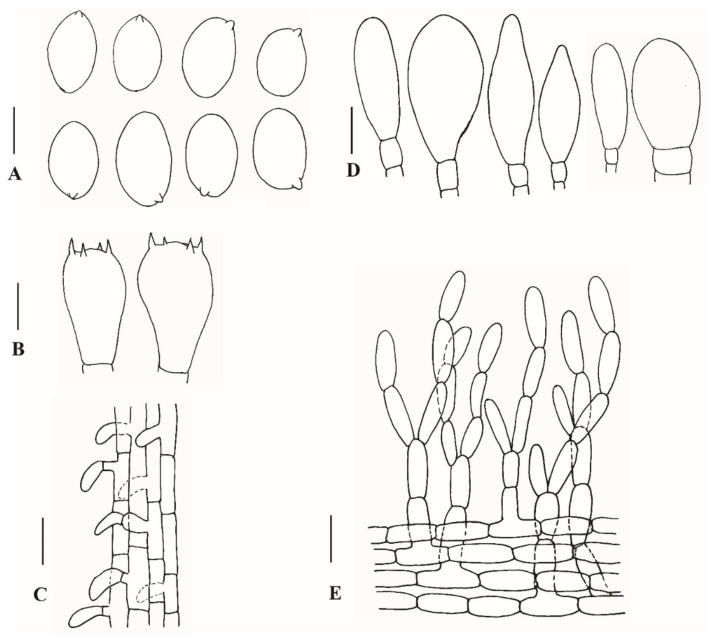
Anatomical features of *Xanthagaricus omanicus* (SQUH-GOO006A). (**A**) Basidiospores; (**B**) Basidia; (**C**) Stipitipellis; (**D**) Cheilocystidia; (**E**) Pileipellis. Scale bars: A = 5 µm; B–D = 10 µm; E = 15 µm.

**Etymology:** “*omanicus*” refers to the country Oman, home of the type locality of the new species (Figure 4b,c and Figure 6).

**Holotype:** Oman, Dhofar, Gogob, 17°21′30.6″ N and 54°11′82.2″ E, under the trees of *Anogeissus dhofarica*, 2 September 2018, SQUH collection GOO-006A (SQUH-GOO006A) GenBank accession: ITS = OM185530).

**Diagnosis:** The diagnostic characteristics of the new species *Xanthagaricus omanicus* are: the largest pileus (70–90 mm diam) among the known species of the genus, which may be convex to applanate, with a slightly depressed center and covered with yellowish to yellowish-green squamules; broadly ellipsoid to ellipsoid, yellowish-brown basidiospores, measuring 7.0–8.5 × 5.0–5.5 µm.

**Description:** Pileus 70–90 mm diam, at young stage parabolic, becoming hemispheric with age, at maturity planoconvex to applanate, with a slightly depressed center; pileus squamulose, squamules orange-yellow (7.5 YR 9/8–10 YR 9/8) to pale orange-yellow (7.5 YR 8/4–7.5 YR 9/4) at the center, towards periphery pale yellowish-green (5 GY 8/4–5 GY 9/4) to moderate yellowish-green (7.5 GY 6/4–7.5 GY 7/4) on a white background; margin revolute with membranous velar remnants. Lamellae free, pale yellowish-brown (10 YR 5/6–10 YR 5/10) to moderate yellowish-brown (10 YR 4/4–10 YR 5/4), broadly ventricose, with a slightly eroded edge, sub-distant to nearly crowded with 3–4 tiers of lamellulae, lamellulae concolorous with lamellae. Stipe 50–60 × 3–5 mm, equal, central, slightly curved, smooth and white above the annulus, slightly pruinose and pale yellowish-brown (5 YR 7/4–10 YR 7/4) below the annulus; annulus not prominent, present as fibrillose velar remnants at the apical zone of stipe, fibrils dark reddish-brown (10 R 3/8–10 R 1/6). Context white, up to 1 mm thick at the center of the pileus; hollow and concolorous with the stipe surface. Odor and taste were not observed. Basidiospores (6.5)7.0–8.5(9.5) × (4.5)5.0–5.5(6.0) µm, on average 7.8 × 5.2 µm, Q = 1.4–1.6, av. Q = 1.5, ellipsoid to broadly ellipsoid or phaseoliform in side view, ellipsoid to oblong in frontal view, thick-walled, smooth, pale yellowish-brown in 5% KOH, without germ pore. Basidia 16.0–19.5 × 9.5–11.5 µm, ellipsoid to clavate, smooth, thin-walled, hyaline, four-spored. Pleurocystidia absent. Cheilocystidia 20.5–29.5 × 9.0–13.0 µm, varying in shape, from narrowly clavate to broadly clavate, ventricose to napiform, abundant. Pileipellis is an irregular tichoderm to intricate trichoderm, consisting of thin-walled, 3.5–7.0 µm wide, subhyaline to pale brown hyphae with some greenish vacuolar pigments, near the septa as observed in KOH; terminal cells measuring 35.0–50.0 × 3.5–7.5 µm. Caulocystidia 11.0−17.5 × 5.0−7.5 µm, clavate to ovate, hyaline, thin-walled. Clamp connections absent.

**Habitat and distribution:** Solitary to scattered in small groups, saprotrophic, on humus-rich soil with dead leaves and wood under trees of *Anogeissus dhofarica*, only known from southern Oman.

**Additional material examined:** Oman, Dhofar, Gogob, under the trees of *Anogeissus dhofarica*, 2 September 2018, SQUH collection GOO-006B (SQUH-GOO006B, GenBank accession: ITS = OM185531).

**Notes:***Xanthagaricus omanicus* is morphologically characterized by the largest pileus among the known species of the genus. Its pileus is covered with yellowish squamules. Microscopically, the basidiospores of the new species are broadly ellipsoid to ellipsoid, yellowish-brown, measuring 7.0–8.5 × 5.0–5.5 µm, and the cheilocystidia are variable in shape, from narrowly clavate to broadly clavate or ventricose to napiform.

Our new species *Xanthagaricus omanicus* and *X. siamensis* Yuan S. Liu and S. Lumyong, recently described from Thailand [10], are morphologically similar by their pileus surfaces which are more or less fibrillose. Macroscopically as well as microscopically, both species can be differentiated. Pileus is comparatively smaller (31–54 mm diam) and covered with grayish-orange to violet-brown fibrils in *X. siamensis*. Basidiospores in *X. siamensis* are substantially smaller (4.0–5.5 × 2.5–3.0 µm) and ellipsoid to oblong. Moreover, on the basis of ML phylogeny, both the species are phylogenetically well differentiated (Figure 3).

In our molecular analyses, *X. omanicus* is shown to be related to *X. necopinatus* and allies. The new species shares basidiospores morphology with *X. necopinatus*, *X. appendiculatus*, *X. pakistanicus,* and *X. purpureosquamulosus*. However, *X. omanicus*, with its large pileus, can be differentiated from these species due to their smaller pilei. Similarly, *X. omanicus* is also related to *X. appendiculatus*. *Xanthagaricus appendiculatus* and *X. omanicus* share similar habitat, and both species occur in the same area around Salalah, Oman. Macroscopically, in *X. omanicus*, the pileus is substantially larger (70–90 mm diam) and covered with appressed, yellowish squamules. Microscopically, the new species can be differentiated on the basis of cheilocystidia. Cheilocystidia in *X. omanicus* are comparatively smaller (20.5–29.5 × 9.0–13.0 µm), variable in shape, from narrowly clavate to broadly clavate, ventricose to napiform. The cystidia in *X. appendiculatus* are larger (26.0–33.0 × 7.0–8.5 µm) and uniformly shaped, i.e., ventricose. The pileus is covered by an irregular epithelium in *X. appendiculatus*, while it is an irregular trichoderm to intricate trichoderm in *X. omanicus*. A detailed comparison of morpho-anatomical features of *X. appendiculatus* and *X. omanicus* with those of other species of the genus is provided in Table 1.

## 4. Discussion

The genus *Xanthagaricus* was introduced by Little Flower et al. [2] with the following characteristics: basidiomata smaller to rarely medium-sized, pileus with distinctive woolly squamules and appendiculate margin; lamellae free, brown, in some cases ink-blue at maturity; stipe cylindrical, almost equal, slightly broader at the apex, annulus rudimentary or absent; spores brown, sometimes yellowish, subglobose to ellipsoid, smooth or slightly ornamented, thick-walled; cheilocystidia present, clavate, subclavate or ventricose; pleurocystidia absent; pileal surface a disrupted epicutis of radial hyphae with plenty of spherical or subspherical cells at the scales; clamp connections absent.

In the *Agaricus* clade of Agaricaceae, *Xanthagaricus* is recovered as a monophyletic group, forming a subclade with *Pseudolepiota*, a monotypic genus recently reported from China [50]. *Pseudolepiota* is differentiated by the white color of the lamellae, hyaline basidiospores, and an ixocutis layer of pileipellis made up of slightly interwoven cylindrical hyphae [50]. However, the synapomorphic characters of the two genera are their pileus squamules, the absence of both pleurocystidia, and clamp connections.

During this study, we collected and described two new species of *Xanthagaricus* from southern Oman. Both the new species *Xanthagaricus appendiculatus* and *X. omanicus* were collected from the Gogob region around Salalah of Dhofar Governorate, Oman. We have examined the distribution of our collection sites following the updated Köppen climate map and classification [32,33]. All the specimens were found in an area with an arid hot desert climate. However, the mountains of Dhofar come under the influence of monsoon, where it usually rains from mid-June to mid-September [34]. This results in dense vegetation and subsequently high diversity of fungal species.

The present study indicates that the mountain and scrublands of southern Oman are rich in mushrooms, and fungal surveys are much needed in the future. This is the first report describing new species of mushrooms from Oman, based on morphology and phylogeny. This study provides a baseline for future scientific studies of these fascinating organisms, which contribute a lot to humanity in terms of food, medicine, pharmacy, etc.

**Key to the Known Taxa of *Xanthagaricus***

1Basidiomata small-sized with pileus (3–15 mm diam)2-Basidiomata small-sized with pileus >15 mm diam132Basidiospores size up to 5 µm in length3-Basidiospores size >5 µm in length93Pileus covered with yellowish or brownish squamules4-Pileus 3–9 mm diam, covered with violet squamules, basidiospores 4.2–4.8 × 2.9–3.4 µm
***X. ianthinus***
4Pileus convex to plano-convex5-Pileus 5–15 mm diam, conico-campanulate, basidiospores 3.8–5 × 2.9–3.6 µm
***X.viridulus***
5Pileus conico-convex or plano-convex6-Pileus 9–12 mm diam, campanulate, basidiospores 4–5 × 3–3.2 µm
***X. ochraceoluteus.***
6Pileus conico-convex, smokey brown or yellowish7-Pileus 4–6 mm diam, ochre-brown, plano-convex, basidiospores 3.7–4.4 × 2.6–3 µm
***X. myriostictus.***
7Pileus brownish, conico-convex, covered with brownish-squamules8-Pileus 10–15 mm diam, brownish, plano-convex, basidiospores 4–5 × 2.7–3.2 µm***X. necopinatus***.8Pileus covered with pale brownish squamules, basidiopsores, subglobse, 4–6 × 4.1–3.8 µm***X. globisporus***.-Pileus with smokey squamules, basidiopsores, ellipsoid, 4.1–4.9 × 2.9–3.4 µm***X. rubescens***.9Pileus plano-convex, covered with yellowish to brownish squamules10-Pileus 10–15 mm diam, with grayish-violet squamules, subhemispherical, basidiospores 5–6 × 3–3.5 µm
***X. caeruleus***
10Pileus with yellowish-brown or violet-brown squamules11-Pileus 10–15 mm diam, greenish squamules, basidiospores 5–5.5 × 3.3–4 µm***X. subepipastus***.11Pileus with yellowish or yellowish-brown squamules12-Pileus 4.5–10 mm diam, violet-brown squamules, basidiospores 4.6–6 × 3.5–4 µm***X. purpureosquamulosus***.12Pileus 5–10 mm diam, convex with yellowish squamules, basidiospores 5–6.3 × 3.5–4.2 µm
***X. gracilis***
-Pileus 8–13 mm diam, with yellowish-brown squamules, basidiospores 5–5.5 × 3–3.5 µm
***X. flavosquamusus.***
13Pileus 15–20 mm diam14-Basidiomata medium-sized, pileus >20 mm diam1814Basidiospores <5.5 µm in length15-Basidiospores >5.5 µm in length1715Pileus convex to campanulate16-Pileus chestnut-brown, conico-convex, basidiospores ellipsoid, 4.3–5.1 × 2.7–3.1 µm
***X.erinaceus.***
16Pileus dark brown, convex, basidiopsores ellipsoid, yellowish, 3.7–4.7 × 2.8–3.4 µm***X. epipastus***.-Pileus campanulate, basidiospores globose to subglobose, 4.2–5 × 2.9–3.5 µm***X. rufomarginatus***.17Pileus conico-convex, covered with yellowish-brown squamules, basidiospores globose to broadly ellipsoid, 7–7.5 × 6–7 µm***X. pakistanicus***.-Pileus plano-convex, covered with purplish squamules, basidiospores ellipsoid to amygdaliform, 6.5–7.5 × 4–5 µm***X. appendiculatus***.18Pileus 20–50 mm diam19-Pileus 70–90 mm diam, applanate, covered with yellowish squamules, basidiospores ellipsoid to broadly ellipsoid, 7–8.5 × 5–5.5 µm***X. omanicus***.19Pileus convex to plano-convex, with brownish squamules20-Pileus conico-campanulate, squamules pale orange, basidiospores ellipsoid to elongate, yellowish-brown, 6–7.5 × 4–4.5 µm
***X. thailandensis.***
20Basidiospores ellipsoid, yellowish to pale-brownish21-Basidiospores ovoid, brownish-yellow2421Basidiospores ellipsoid with truncate base or oblong22-Basidiospores ellipsoid with regular base, yellowish or brownish2322Basidiospores ellipsoid to oblong, yellowish-brown, 4–5.5 × 2.5–3 µm
***X. siamensis.***
-Basidiospores ellipsoid with truncate base, brownish-yellow, 5–6 × 3.4–4.2 µm***X. luteolosporus***.23Basidiospores golden-yellow, 6–7.4 × 4–4.8 µm***X. chrysosporus***.-Basidiospores brownish-yellow, 5–5.5 × 3–4 µm***X. taiwanensis***.24Basidiospores ovoid with truncate base, yellowish-brown, 4.2–5 × 2.9–3.5 µm***X. subaeruginosus***.-Basidiospores without trancate base, brownish-yellow, 4.9–5.8 × 3.7–4.3 µm***X. calicutensis***.

## Figures and Tables

**Table 1:** Characteristics distinguishing *Xanthagaricus appendiculatus* and *X. omanicus* from the other *Xanthagaricus* species.

Species	Pileus Diameter (mm)	Stipe Size (mm)	Basidiospores Size (µm)	Squamules Cells/Size (µm)	References
*Xanthagaricus appendiculatus* sp. nov.	15–20	40–60 × 2–4	6.5–7.5 × 4.0–5.0	Irregular epithelium/13.5–21.5	Examined in the present study.
*X. calicutensis*	30–40	45 × 4	4.9–5.8 × 3.7–4.3	Sub-hymeniform/10–20	[1]
*X. caeruleus*	10–15	22–35 × 1.5–2	5–6 × 3–3.5	Pseudoparenchymatous/9–21 × 9–15	[5]
*X. chrysosporus*	20–30	30 × 2	6.0–7.4 × 4.0–4.8	Sub-hymeniform/11–14	[1]
*X. epipastus*	15–20	40 × 2	3.7–4.7 × 2.8–3.4	Sub-hymeniform/10–27	[1]
*X. erinaceus*	15–20	30 × 1	4.3–5.1 × 2.7–3.1	Hymeniform/10–24 × 6–8	[1]
*X. flavidorufus*	20–27	70 × 1	4.4–5.4 × 3.0–3.5	Vesiculate/10	[1,2]
*X. flavosquamosus*	8–13	20–30 × 1.5–2	5–5.5 × 3–3.5	Epithelium/6–12 × 6–10	[14]
*X. globisporus*	10	20 × 1	4.6–5.1 × 4.1–4.8	Hymeniform, pseudoparenchymatous/10–25 × 8–25	[1]
*X. gracilis*	5–10	30–35 × 1	5.0–6.3 × 3.5–4.2	Subhymeniform/8–22 × 8–14	[1]
*X. ianthinus*	3–9	12–24 × 0.8–1.5	4.2–4.8 × 2.9–3.4	Epithelium/7.6–20.7 × 5.4–15.8	[15]
*X. luteolosporus*	30–40	50 × 4	5.0–6.0 × 3.4–4.2	Hymeniform/7–30	[1]
*X. myriostictus*	4–6	16 × 1	3.7–4.4 × 2.6–3.0	Hymeniform to pseudoparenchymatous/9–20	[1]
*X. necopinatus*	10–15	18–28 × 1.5–2	4–5 × 2.7–3.2	Epithelium/9–15 × 6–10	[5]
*X. ochraceoluteus*	9–12	30 × 1	4.0–5.2 × 3.0–3.2	Pseudoparenchymatous/5–15	[13]
*X. omanicus* sp. nov.	70–90	50–60 × 3–5	7.0–8.5 × 5.0–5.5	Intricate trichoderm/3.5–7.0	Examined in the present study.
*X. pakistanicus*	10–20	10–15 × 1–2	7.0–7.5 × 6.5–7	Pseudoparenchymatous epithelium/20–47 × 18–44	[6]
*X. purpureosquamulosus*	4.5–10	15–30 × 1–1.5	4.5–6 × 3.5–4	Irregular epithelium/7.5–25	[11]
*X. rubescens*	10–16	20 × 1	4.1–4.9 × 2.9–3.4	Pseudoparenchymatous/15–35 × 8–12	[1]
*X. rufomarginatus*	20	20 × 3	4.0–4.2 × 2.5–3.0	Pseudoparenchymatous epithelium/7–17 × 5–10	[12]
*X. siamensis*	31–54	35–65 × 2–6	4.0–5.5 × 2.5–3.0	Cutis/3.5–8	[10]
*X. subaeruginosus*	45	40(90) × 2	4.2–5.0 × 2.9–3.5	Pseudoparenchymatous to hymeniform/22–40 × 14–35	[1]
*X. subepipastus*	10–15	15–20 × 1	4.8–5.3 × 3.3–3.9	Subhymeniform to pseudoparenchymatous/11–20	[1]
*X. taiwanensis*	15–35	40–70 × 6–9	5.0–5.5 × 3.0–4.0	Pseudoparenchymatous/10–20 × 8–12	[7]
*X. thailandensis*	30−45	35−55 × 5−8	6−7.5 × 4−4.5	Pseudoparenchymatous/5−26 × 4.5−8.5	[9]
*X. viridulus*	5–15	10–25 × 1	3.8–5.0 × 2.9–3.6	Pseudoparenchymatous to hymeniform/22–40 × 14–35	[1]

## Data Availability

Publicly available datasets were analyzed in this study. The resulting alignment was deposited in TreeBASE (http://www.treebase.org; accession number S29213 (accessed on 8 January 2022)). All newly generated sequences were deposited in GenBank (https://www.ncbi.nlm.nih.gov/genbank/ (accessed on 8 January 2022); mentioned in the text and in Figure 3). All new taxa were deposited in MycoBank (https://www.mycobank.org/ (accessed on 8 January 2022); MycoBank identifiers follow new taxa).
